# Biannual Differences in Interest Peaks for Web Inquiries Into Ear Pain and Ear Drops: Infodemiology Study

**DOI:** 10.2196/28328

**Published:** 2021-06-24

**Authors:** Faris F Brkic, Gerold Besser, Martin Schally, Elisabeth M Schmid, Thomas Parzefall, Dominik Riss, David T Liu

**Affiliations:** 1 Department of Otorhinolaryngology, Head and Neck Surgery Medical University of Vienna Vienna Austria

**Keywords:** otitis media, otitis externa, otalgia, Google Trends, infodemiology, infoveillance, infodemic, social listening

## Abstract

**Background:**

The data retrieved with the online search engine, Google Trends, can summarize internet inquiries into specified search terms. This engine may be used for analyzing inquiry peaks for different medical conditions and symptoms.

**Objective:**

The aim of this study was to analyze World Wide Web interest peaks for “ear pain,” “ear infection,” and “ear drops.”

**Methods:**

We used Google Trends to assess the public online interest for search terms “ear pain,” “ear infection,” and “ear drops” in 5 English and non–English-speaking countries from both hemispheres based on time series data. We performed our analysis for the time frame between January 1, 2004, and December 31, 2019. First, we assessed whether our search terms were most relevant to the topics of ear pain, ear infection, and ear drops. We then tested the reliability of Google Trends time series data using the intraclass correlation coefficient. In a second step, we computed univariate time series plots to depict peaks in web-based interest. In the last step, we used the cosinor analysis to test the statistical significance of seasonal interest peaks.

**Results:**

In the first part of the study, it was revealed that “ear infection,” “ear pain,” and “ear drops” were the most relevant search terms in the noted time frame. Next, the intraclass correlation analysis showed a moderate to excellent reliability for all 5 countries’ 3 primary search terms. The subsequent analysis revealed winter interest peaks for “ear infection” and “ear pain”. On the other hand, the World Wide Web search for “ear drops” peaked annually during the summer months. All peaks were statistically significant as revealed by the cosinor model (all *P* values <.001).

**Conclusions:**

It can be concluded that individuals affected by otitis media or externa, possibly the majority, look for medical information online. Therefore, there is a need for accurate and easily accessible information on these conditions in the World Wide Web, particularly on differentiating signs and therapy options. Meeting this need may facilitate timely diagnosis, proper therapy, and eventual circumvention of potentially life-threatening complications.

## Introduction

Google Search (Google LLC) is the most commonly used internet search engine, and almost two-thirds of all daily online inquiries are performed using this platform. [1] Google Trends (GT) delivers information on geographical and temporal patterns of search volumes for inquiries performed with Google. [2] Notably, several studies have already used GT to analyze web user searches for different otolaryngological symptoms, conditions, and diseases. [3-6] Furthermore, conditions in other medical disciplines have been assessed, including in cardiology [7,8], urology [9], and orthopedic surgery [10]. In general, the internet has become an appealing source of health care information, particularly for younger people, who are more amenable to new and innovative approaches for gaining novel information in an online environment [11]. Therefore, internet search volumes for certain medical conditions could potentially follow their annual incidence peaks.

Acute otitis media (OM) and otitis externa (OE) are common conditions in otolaryngology and represent the middle ear and external auditory canal’s inflammation, respectively [12,13]. OM is mostly a result of a dysfunction of the auditive tube due to upper airway tract infections [12]. New diagnostic guidelines recommend combining medical history, clinical symptoms, and strict otoscopic criteria to determine this diagnosis [14]. These include moderate or severe tympanic membrane bulging combined with otorrhea or mild bulging combined with recent onset of ear pain. OE’s incidence is comparable to that of OM and affects up to 10% of people at some point in life. [13] Some common symptoms tend to overlap with those of OM, such as ear pain or otorrhea. Nevertheless, some critical differentiation signs exist, such as ear itching and tenderness of the tragus or pinna, which are OE-specific symptoms [15]. Based on the many similarities between these two conditions, it may be assumed that individuals tend to misdiagnose or incorrectly treat themselves by referring to information available online.

It has been noted that OM occurs mostly in the winter months, which correlates with the incidence rates of upper airway infection [12,16-19]. In contrast, OE incidence rises significantly during the warmer summer months, mostly due to increased humidity, sweating, and swimming (ie, water exposure to the outer ear canal [15,20,21]). Interestingly, a study on seasonal peaks of acute OM incidence revealed peaks in the winter and summer months [22].

To the best of our knowledge, only one study group has assessed online user behavior regarding otologic conditions. Specifically, they correlated GT search for “ear drops” with Medicaid prescription frequency for ototopical agents [23]. However, to date, no study has analyzed World Wide Web public inquiries into other OM- and OE-related terms (such as ear pain or ear infection). Therefore, our study aimed to analyze the web-based interest into ear pain, ear infections, and the associated treatment options (ear drops). This study’s results may help gain novel insights into the temporal frequency of internet searches into ear pain–related search terms and clarify the temporal dynamics of the affected individuals who search for symptoms or treatment options regarding these medical conditions.

## Methods

GT was used to explore the online search interest for “ear pain” and related terms entered into the Google search engine from various countries worldwide. The relative search volume (RSV) shows user interest in specific search terms. It ranges from 0 to 100 (higher interest correlates with higher score). The normalization steps are described elsewhere [24]. We explored RSV for the following 3 search terms (and the country-specific translation) related to ear infections: “ear drops” (“Ohrentropfen”), “ear pain” (“Ohrenschmerzen”), and “ear infection” (“Ohr Entzündung”). In order to cover both hemispheres and 2 languages (English and German), we assessed internet-based inquiries in the following countries: Australia, Canada, Germany, the United States, and the United Kingdom. The data were retrieved in the “Health” category for the time frame between January 1, 2004, and December 31, 2019.

The 3 preliminary search terms were entered on August 18, 2020, and the function “Related (Top)” was used to show search terms related to the preliminary search terms in every analyzed country. We ensured that we analyzed 3 search terms with the highest RSV in the context of “ear pain,” “ear infection,” and “ear drops” by using the GT function “Comparison.” We therefore compared the RSV of all related search terms against the 3 preliminary search terms. The most relevant search terms associated with each of the 3 preliminary search terms were used for further data acquisition and analyses. Furthermore, we also added the search terms “otitis” and “otitis media” (“Gehörgangsentzündung” and “Mittelohrentzündung”, respectively, for Germany) to cover the full spectrum of ear pain–related search terms.

We then entered each of the aforementioned 5 ear pain–related search terms (ie, “ear drops,” “ear pain,” “ear infection,” “otitis,” and “otitis media”) on April 25, 2021, and used the function “Worldwide” to assess regional differences in country-specific RSV. We specified our searches for the “Health” category and the time frame between January 1, 2004, and December 31, 2019. Finally, a reliability analysis was performed by downloading selected search terms for 10 consecutive days, starting from August 18, 2020. It has previously been reported that slightly different results are shown if GT data are downloaded on different days [3,16].

The analysis was performed with R software version 3.5.1 (R Foundation for Statistical Computing) with the “season” and “psych” packages. The visualization of these data was performed with Prism 9.0.0 software (GraphPad). The intraclass correlation coefficient (ICC; 2-way random model) was used for examination of GT data reliability. Poor reliability was defined as an ICC lower than 0.4, moderate reliability as an ICC higher than or equal to 0.4 and lower than 0.75, good reliability as an ICC higher than or equal to 0.75 and lower than 0.9, and excellent reliability as an ICC greater than or equal to 0.9. The cosinor regression model was used to detect seasonality in time series data. The exact model is described in detail elsewhere [25]. In short, the cosinor model is a parametric model that captures seasonal patterns using a sinusoid. We fitted an annual seasonal pattern to our time series data and therefore defined the annual seasonal cycle as 12 (months). As the cosinor model assumes the sinusoid to be symmetric and stationary, a peak (P, peak) and a nadir point (L, low point, defined as peak month +/–6 months) are defined once per year. The cosinor analysis also computes an amplitude (size), which represents the magnitude of the seasonal effect. As the sinusoid is described by both a sine and a cosine term, statistical significance can be tested as part of a generalized linear model. We set the *P* value to .03 to adjust for multiple comparisons (sine and cosine term).

The visualization of worldwide country-specific differences in RSV for our 5 ear pain–related search terms (mentioned previously) was performed with Python 3.9.4 [26] in combination with the libraries NumPy [27], Pandas [28], Matplotlib [29], and Geopandas [30].

For the current study, only publicly available and nonidentifiable data were explored. Therefore, according to the guidelines of the institutional review board of the Medical University of Vienna, study approval was not needed.

## Results

### Search Terms Related to Ear Infection, Ear Pain, and Ear Drops

As we wanted to perform the analysis only using search terms with the highest RSV, our first step was to assess if preliminary search terms were in fact the most searched in regard to the following 3 terms: (1) “ear pain,” (2) “ear infection,” and (3) “ear drops.” We therefore entered each of the 3 primary search terms into GT for the time frame between January 1, 2004, and December 31, 2019. The search was performed using the “Health” category. Furthermore, the GT function “Related queries (Top)” was used to identify search terms that users entered on GT following the specified one. This step was followed by comparing the RSV of each related search term with each of the 3 preliminary search terms to evaluate whether the latter terms had the highest RSV within their respective category. It was indeed shown that our 3 preliminary search terms (“ear pain,” “ear infection,” and “ear drops”) represented the 3 most relevant search terms within their respective category in all English-speaking countries. Only the search term “Mittelohrentzündung” (middle ear infection) had a higher RSV compared to “Ohr Entzündung” (ear infection) in Germany (Tables S1-S5, Multimedia Appendix 1). To cover the full spectrum of ear pain–related search terms, we also added “otitis” and “otitis media” (“Gehörgangsentzündung” and “Mittelohrentzündung” for Germany) for further data analyses.

### Regional Differences in Relative Search Volume for Ear Infection–Related Search Terms

In the next step, we assessed country-specific differences in RSV for our 5 ear infection–related search terms: “ear pain,” “ear infection,” “ear drops,” “otitis,” and “otitis media.” We used the GT function “Worldwide” to compare the country-specific RSVs.

**Figure 1:**
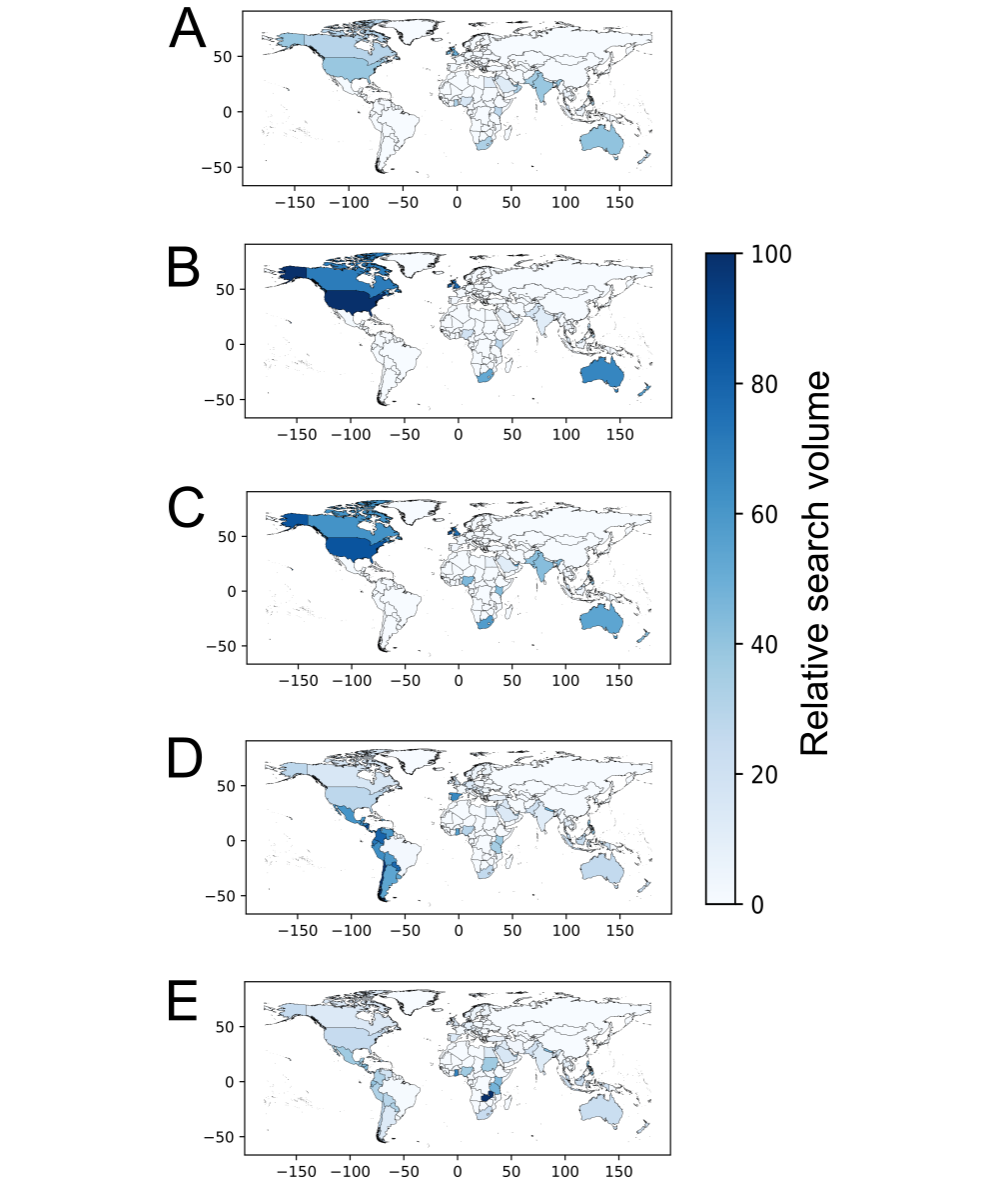
World maps showing country-specific variations in relative search volume for (A) “ear drops,” (B) “ear infection,” (C) “ear pain,” (D) “otitis,” and (E) “otitis media.” The color legend represents the relative search volume. The x-axis and the y-axis represent the longitude and latitude, respectively. The maps have been created with the GeoPandas library [30].

The graphical analysis revealed regional differences in RSV for each of the 5 ear infection–related search terms. “Ear infection” and “ear pain” were searched more often in English-speaking countries from the Northern Hemisphere, while “otitis” and “otitis media” were searched more often in countries from the Southern Hemisphere (Figure 1).

### Reliability of Ear Infection–Related Search Terms

We performed the reliability analysis using the ICC for GT time series data of our 3 final search terms extracted on 10 consecutive days, starting on August 18, 2020.

The 3 primary search terms showed good to excellent reliability in English-speaking countries, with correlation coefficients ranging between 0.78 and 0.97 for “ear drops,” between 0.87 and 0.99 for “ear infection,” and between 0.89 to 0.99 for “ear pain.” Furthermore, reliability analyses in Germany also revealed excellent correlation coefficients for 2 of the 3 primary search terms: ICC of 0.91 for “Ohrentropfen” (ear drops), ICC of 0.47 for “Ohr Entzündung” (ear infection), and ICC of 0.98 for “Ohrenschmerzen” (ear pain; Table 1). The analysis revealed less reliable results for our 2 additional search terms. The ICC ranged between 0.36 and 0.86 for “otitis” and between 0.37 to 0.74 for “otitis media” in the English-speaking countries. Further correlation analyses revealed an ICC of 0.93 for “Mittelohrentzündung” and an ICC of 0.75 for “Gehörgangsentzündung” in Germany.

**Table 1:** Reliability of single and averaged time series data on “ear drops,” “ear infection,” “ear pain,” “otitis,” and “otitis media” in all countries.

Search term by country				ICC^a^		Lower bound^b^		Upper bound^b^	*F* test^c^		Df1^d^		Df2^e^		*P* value
**Australia**															
	**Ear drops**														
		Single^f^	0.78		0.74		0.81		35.96	191		1719		<.001	
		Average^g^	0.97		0.97		0.98		35.96	191		1719		<.001	
	**Ear infection**														
		Single	0.87		0.84		0.89		71.81	191		1719		<.001	
		Average	0.99		0.98		0.99		71.81	191		1719		<.001	
	**Ear pain**														
		Single	0.89		0.87		0.91		88.27	191		1719		<.001	
		Average	0.99		0.99		1.00		88.27	191		1719		<.001	
	**Otitis**														
		Single	0.36		0.27		0.44		8.90	191		1719		<.001	
		Average	0.85		0.79		0.89		8.90	191		1719		<.001	
	**Otitis media**														
		Single	0.46		0.39		0.54		11.70	191		1719		<.001	
		Average	0.90		0.87		0.92		11.70	191		1719		<.001	
**Canada**															
	**Ear drops**														
		Single	0.83		0.80		0.86		52.04	191		1719		<.001	
		Average	0.98		0.98		0.98		52.04	191		1719		<.001	
	**Ear infection**														
		Single	0.87		0.84		0.89		74.41	191		1719		<.001	
		Average	1.99		0.98		0.99		74.41	191		1719		<.001	
	**Ear pain**														
		Single	0.92		0.89		0.93		123.40	191		1719		<.001	
		Average	0.99		0.99		0.99		123.40	191		1719		<.001	
	**Otitis**														
		Single	0.86		0.83		0.89		73.57	191		1719		<.001	
		Average	0.98		0.98		0.99		73.57	191		1719		<.001	
	**Otitis media**														
		Single	0.47		0.41		0.53		10.57	191		1719		<.001	
		Average	0.90		0.87		0.92		10.57	191		1719		<.001	
**United Kingdom**															
	**Ear drops**														
		Single	0.97		0.96		0.97		300.23	191		1719		<.001	
		Average	1.00		1.00		1.00		300.23	191		1719		<.001	
	**Ear infection**														
		Single	0.98		0.98		0.99		765.13	191		1719		<.001	
		Average	1.00		1.00		1.00		765.13	191		1719		<.001	
	**Ear pain**														
		Single	0.99		0.98		0.99		851.84	191		1719		<.001	
		Average	1.00		1.00		1.00		851.84	191		1719		<.001	
	**Otitis**														
		Single	0.33		0.18		0.47		17.05	191		1719		<.001	
		Average	0.83		0.68		0.90		17.05	191		1719		<.001	
	**Otitis media**														
		Single	0.37		0.24		0.48		13.00	191		1719		<.001	
		Average	0.85		0.76		0.90		13.00	191		1719		<.001	
**United States**															
	**Ear drops**														
		Single	0.97		0.97		0.98		404.42	191		1719		<.001	
		Average	1.00		1.00		1.00		404.42	191		1719		<.001	
	**Ear infection**														
		Single	0.99		0.99		0.99		1096.64	191		1719		<.001	
		Average	1.00		1.00		1.00		1096.64	191		1719		<.001	
	**Ear pain**														
		Single	0.99		0.99		0.99		1523.05	191		1719		<.001	
		Average	1.00		1.00		1.00		1523.05	191		1719		<.001	
	**Otitis**														
		Single	0.51		0.36		0.64		25.50	191		1719		<.001	
		Average	0.91		0.85		0.95		25.50	191		1719		<.001	
	**Otitis media**														
		Single	0.74		0.70		0.79		32.61	191		1719		<.001	
		Average	0.97		0.96		0.97		32.61	191		1719		<.001	
**Germany**															
	**Mittelohrentzündung**														
		Single	0.93		0.91		0.94		132.45	191		1719		<.001	
		Average	0.99		0.99		0.99		132.45	191		1719		<.001	
	**Ohrenschmerzen**														
		Single	0.98		0.98		0.99		691.66	191		1719		<.001	
		Average	1.00		1.00		1.00		691.66	191		1719		<.001	
	**Ohrentropfen**														
		Single	0.91		0.89		0.92		98.68	191		1719		<.001	
		Average	0.99		0.99		0.99		98.68	191		1719		<.001	
	**Ohr Entzündung**														
		Single	0.47		0.38		0.55		12.93	191		1719		<.001	
		Average	0.90		0.86		0.92		12.93	191		1719		<.001	
	**Gehörgangsentzündung**														
		Single	0.75		0.71		0.79		32.38	191		1719		<.001	
		Average	0.97		0.96		0.98		32.38	191		1719		<.001	

^a^ICC: intraclass correlation coefficient.

^b^lower and upper bound = 95% confidence interval of the intraclass correlation coefficient.

^c^*F* test for significance of the correlation coefficient.

^d^Df1: numerator degrees of freedom.

^e^Df2: denominator degrees of freedom.

^f^Single: single time series data.

^g^Average: averaged time series data.

### Biannual Differences in Web-Based Interest Peaks for Our Ear-Related Search Terms

**Figure 2:**
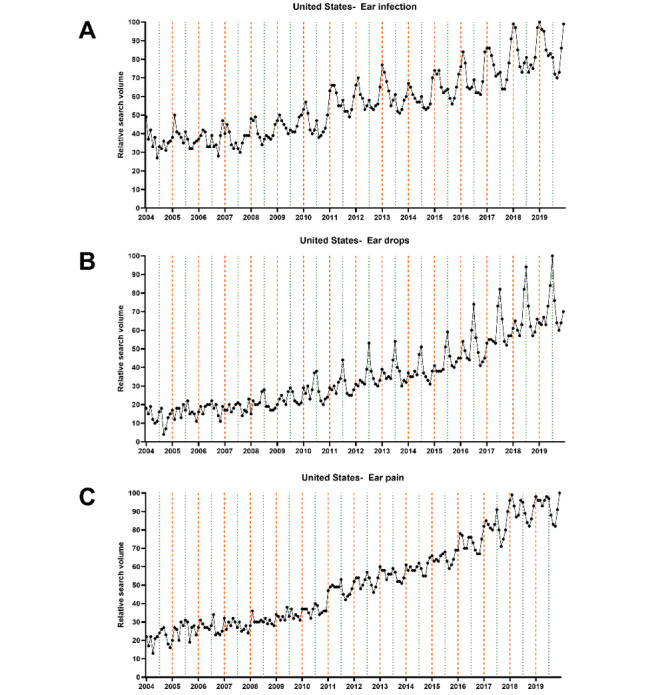
Univariate time series plot showing monthly changes in relative search volume for (A) “ear infection,” (B) “ear drops,” and (C) “ear pain” in the United States between January 2004 and December 2019. The thick vertical orange lines mark January 1, while the thin vertical green lines mark July 1. The black points represent the relative search volume of each of the 12 months.

**Figure 3:**
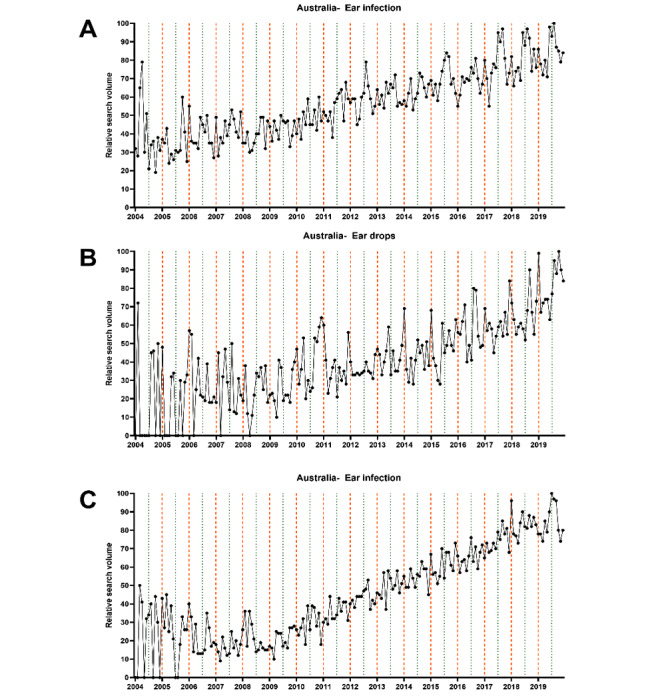
Univariate time series plot showing monthly changes in relative search volume for (A) “ear infection,” (B) “ear drops,” and (C) “ear pain” in Australia between January 2004 and December 2019. The thick vertical orange lines mark January 1, while the thin vertical green lines mark July 1. The black points represent the relative search volume of each of the 12 months.

**Figure 4:**
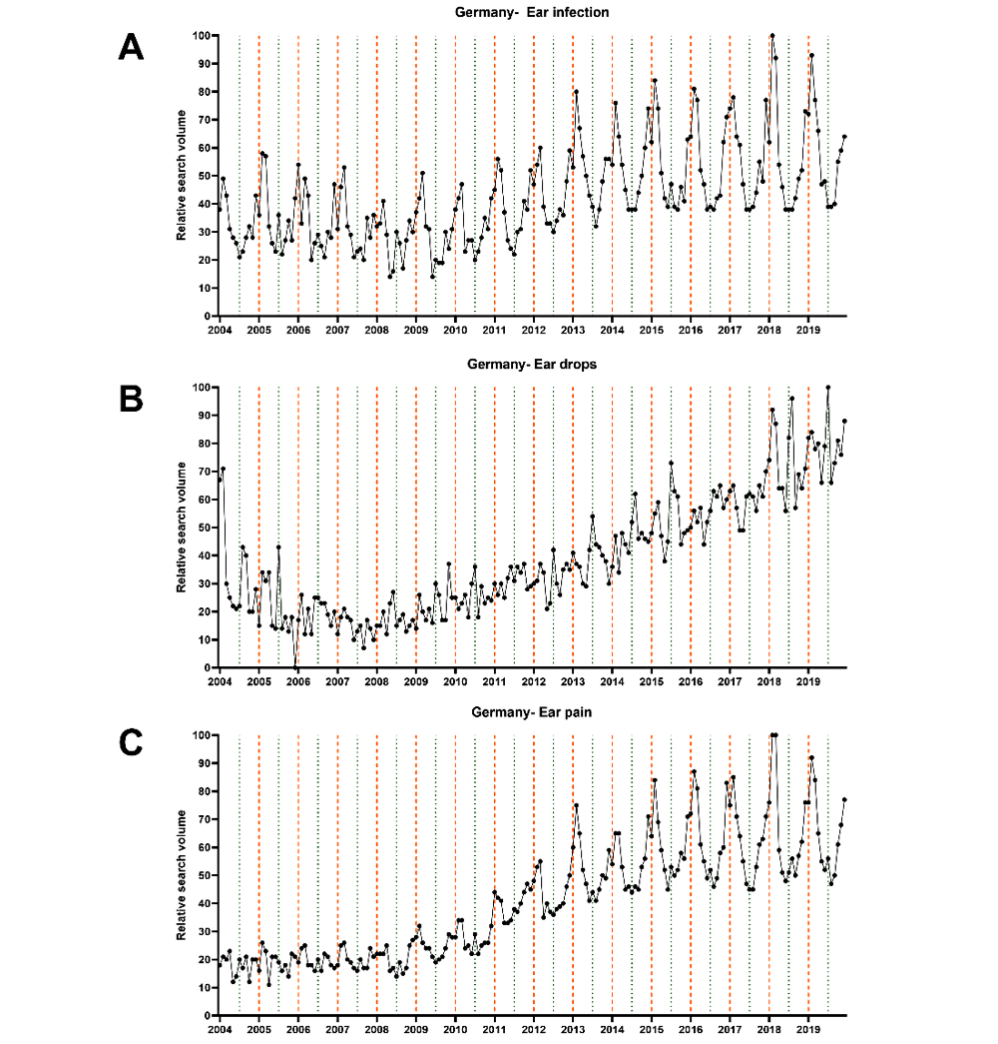
Univariate time series plot showing monthly changes in relative search volume for (A) “ear infection,” (B) “ear drops,” and (C) “ear pain” in Germany between January 2004 and December 2019. The thick vertical orange lines mark January 1, while the thin vertical green lines mark July 1. The black points represent the relative search volume of each of the 12 months.

Next, we performed a time series analysis to depict the seasonal variations graphically and statistically. The data sets extracted on the first day of data retrieval (August 18, 2020) were used for this analysis. We computed univariate time series plots to analyze monthly changes in RSV graphically (Figures 2-4). Subsequently, the cosinor model was used to analyze the time series data to depict peaks and nadirs in web-based interest for each of the 3 final search terms.

**Figure 5:**
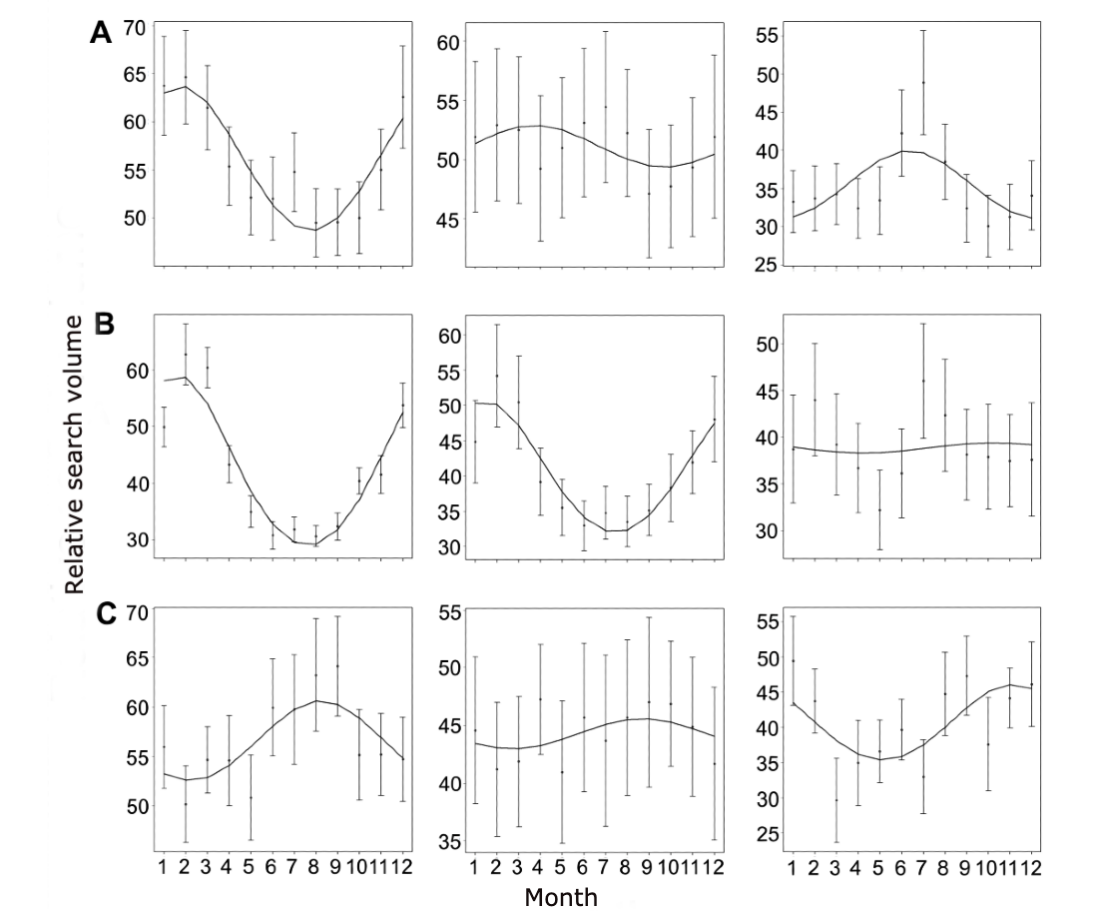
Cosinor analysis plots showing monthly variations in relative search volume for “ear infection,” “ear pain,” and “ear drops” (from left to right) in (A) the United States, (B) Germany, and (C) Australia. The black dots mark the monthly mean relative search volume, while the error bars mark the SE. The numbers on the x-axis represent the corresponding months (ie, 1 = January, 2 = February).

The graphical analysis revealed interest peaks in the summer months for “ear drops” in English- and non–English-speaking countries from both hemispheres (Figure 5). In contrast, peaks in web-based interest for “ear infection” and “ear pain” were apparent in the winter months. Subsequent cosinor analyses revealed all of these peaks to be significant (all *P* values <.001) (Table 2).

**Table 2:** Cosinor analysis on seasonality of “ear drops,” “ear pain,” “ear infection,” “otitis,” and “otitis media”.

Term by country		Amplitude	Peak^a^	Nadir^a^	SE	*P* value
**Australia**						
	Ear drops	5.73	11.1	5.1	0.016	<.001
	Ear pain	1.32	8.7	2.7	0.015	<.001
	Ear infection	4.20	8.2	2.2	0.014	<.001
	Otitis	3.67	6.0	12.0	0.022	<.001
	Otitis media	3.82	6.9	12.9	0.023	.002
**Canada**						
	Ear drops	0.91	7.3	1.3	0.018	<.001
	Ear pain	1.68	2.1	8.l	0.015	<.001
	Ear infection	9.77	1.5	7.5	0.013	<.001
	Otitis	11.13	1.2	7.2	0.015	<.001
	Otitis media	6.44	2.0	8.0	0.018	<.001
**United Kingdom**						
	Ear drops	2.63	7.6	1.6	0.016	<.001
	Ear pain	2.31	12.3	6.3	0.017	<.001
	Ear infection	4.59	12.5	6.5	0.016	<.001
	Otitis	3.90	2.0	8.0	0.015	<.001
	Otitis media	7.76	1.4	7.4	0.018	<.001
**United States**						
	Ear drops	4.74	6.4	12.4	0.017	<.001
	Ear pain	1.79	3.7	9.7	0.014	<.001
	Ear infection	8.03	1.8	7.8	0.014	<.001
	Otitis	3.18	1.7	7.7	0.011	<.001
	Otitis media	8.07	1.7	7.7	0.012	<.001
**Germany**						
	Mittelohrentzündung	17.78	1.6	7.6	0.016	<.001
	Ohrenschmerzen	10.36	1.5	7.5	0.016	<.001
	Ohrentropfen	0.55	10.3	4.3	0.016	<.001
	Ohr entzündung	1.98	8.9	2.9	0.025	<.001
	Gehörgangsentzündung	12.72	8.0	2.0	0.018	<.001

^a^The values in this column correspond to the respective month (eg, 1=January, 2=February).

## Discussion

The current study used GT and revealed winter and summer peaks in online search volumes for OE- and OM-related search terms, which corresponded with the winter and summer incidence rise of these conditions. In particular, highly reliable results revealed that the search volume for “ear drops” peaked in the summer months, potentially reflecting the interest in OE at this time. On the contrary, the annual increase in search frequency for terms “ear pain” and “ear infection” (hypothetically reflecting both OE and OM) was observed during the winter months. This discrepancy between online search peaks could indicate a potential mistake in treatment by self-diagnosis or self-treatment of either of these two conditions by patients searching for therapy options online. As noted, one study already assessed this problem by correlating Google search frequency for ear drops and Medicaid prescriptions for ototopical agents [23]. That study, however, included some methodological limitations: no further GT analysis regarding related terms or symptoms of OM or OE was performed, the reliability of the GT data was not analyzed, and the online search frequency for noted terms was assessed for only 1 country. Therefore, the significance of our study is mainly its highly reliable GT data, reproducibility in different countries from both hemispheres, and significant interest peaks revealed by the cosinor analysis for all 3 search terms.

As noted, GT has often been used to analyze user-associated online behavior in regard to different otorhinolaryngologic conditions [3-6,16]. Furthermore, public online interest for certain oral, maxillofacial, and dentistry problems has been analyzed by several authors [31-33]. Moreover, this engine was used for assessing public inquiries into seasonal influenza [34,35]. Even the currently prevailing and life-changing COVID-19 pandemic was analyzed [36,37], and authors have also discussed using GT to predict COVID-19 outbreaks [38,39].

There are several critical similarities between OM and OE regarding symptoms and therapy options. The overlapping symptoms include ear pain and otorrhea [14,15], while specific OE signs include an itchy ear and tragus pain [15]. Therapy regimens for both conditions include analgesics, but the indication for antibiotic therapy varies significantly. Although bacteria can be isolated from the middle ear in 50% to 90% of cases [40,41], not all OM patients require antibiotic therapy at initial presentation. Clinical observation and analgesic therapy are the first-line treatment approaches in adult patients and children over 6 years with mild symptoms. However, either a clinical follow-up within 2 to 3 days should be scheduled, or a backup antibiotic should be prescribed and used in cases where symptoms persist [14,42]. Regarding OE, therapy options include ear cleaning, analgesics, and topical treatment (antibiotics, steroids, antiseptics, antifungals, or combinations) [43]. Oral antibiotics are not indicated for treatment of OE, as they prolong time to clinical cure and are not associated with better outcomes as compared with a topical agent used alone in uncomplicated cases [20,44]. Additionally, the overuse of systemic antibiotics contributes to the global problem of antibiotic resistance [16]. In summary, systemic or oral antibiotic is only required in severe and persistent OM, while OE can be treated mostly with topical therapy using ear drops.

Incompletely treated or untreated OM can result in complications, such as mastoiditis, facial nerve paresis or palsy, or labyrinthitis. Therefore, symptoms of OM warrant further diagnosis and therapeutic approaches [45]. Furthermore, these can occur in cases with inadequately treated OM (eg, in cases of antibiotic-resistant bacteria) [45]. The most typical symptoms of acute mastoiditis are a retroauricular swelling and a protruded pinna. Other clinical signs include retroauricular erythema and pain, or an abscess of the external auditory canal [46,47]. Timely recognition of these signs and a prompt referral to an otolaryngology department are crucial in treating OM complications. The next steps include intravenous antibiotic therapy and a computer tomography scan in cases that do not respond to therapy [48]. Surgical treatment such as mastoidectomy remains the most efficient therapy option for patients with intracranial complications or mastoid abscess [49]. Malignant (necrotizing) OE is a potentially fatal complication of acute OE and affects older adults, immunocompromised individuals, and patients with undertreated diabetes mellitus [50]. The most common signs are otalgia, subacute hearing loss, and intensive otorrhea [50]. The infection can extend to the mastoid and the skull base and can potentially result in facial nerve palsy, venous sinus thrombosis, osteomyelitis, or even death [51]. Although patients potentially self-manage these conditions by looking for information online, timely referral to the hospital appears crucial, as a definitive diagnosis can only be made by a medical professional. Not postponing a doctor’s appointment can lead to proper treatment and circumvention of potentially life-threatening complications.

OM affects all age groups, but about 80% of children have at least 1 acute OM episode before school age [12]. However, a plethora of pediatric patients does not necessarily warrant antibiotic therapy. Systemic antibiotics should be routinely indicated only in children above the age of 6 months with severe symptoms and in children older than 2 years with bilateral acute OM [14]. Observation with scheduled clinical follow-up is recommended in children 2 to 12 years old with nonsevere symptoms [14]. Furthermore, OM with effusion is often misdiagnosed as acute OM and overtreated with antibiotics [52]. Overtreatment is also a common problem affecting patients with OE. One study group noted that about 20% to 40% of patients with an acute OE are primarily and unnecessarily treated with a systemic antibiotic [53] even though systemic antibiotics do not necessarily produce better clinical outcomes [20]. In summary, the timely differentiation between OM and OE is crucial, as it can result in proper therapy, reduction of overtreatment, and circumvention of possibly life-threatening complications.

The current study faces limitations associated with its infodemiological design. Potential standard limitations of infodemiological studies could influence the interpretation of our results. As we used a single search engine to retrieve all data, selection bias is a potential limitation. However, about two-thirds of daily internet searches are performed using Google web search, which minimizes this risk [1]. A further selection bias is reflected by the fact that people from higher-income areas have access and tend to use the internet for any kind of information, particularly regarding certain medical conditions [54]. Furthermore, with GT, the data on the age or gender of users who search for different terms cannot be assessed. This limitation could be a confounding factor, as younger people are more likely to use online search engines to gather information on medical conditions [55]. On the other hand, studies designed around using infodemiological methods can arguably be more extensive, detailed, and real time than epidemiological studies. Therefore, with these methods, the information retrieval and the efficacy of the research can be improved. Furthermore, it can only be assumed that RSV varies only slightly across individual areas within each country. We included larger countries in our analysis. Thus, our results may only represent those regions with more inhabitants (and therefore higher RSV). Future studies will reveal whether regional differences have a significant influence on online search frequency for different medical conditions.

In conclusion, we observed biannual (summer and winter) peaks in searches for otitis externa and media and related terms, which correlated with the respective annual incidence increase of these two conditions. These findings thus underline the necessity for accurate and easily accessible medical information on the internet, particularly for diagnosis, appropriate therapy options, and differentiating between OE and OM. This type of information may reduce overtreatment with antibiotics in OE cases and mitigate the global problem of antibiotic resistance. Finally, prompt and early detection of potentially life-threatening complications and subsequent further diagnosis and therapy could be facilitated.

## Appendix

Multimedia Appendix 1 Results from relative search volume comparison between primary and related search terms in Australia.

## Abbreviations

GT: Google Trends
ICC: intraclass correlation coefficient
OE: otitis externa
OM: otitis media
RSV: relative search volume

## References

[1] Browser market share. Market Share Statistics for Internet Technologies.

[2] Pier MM, Pasick LJ, Benito DA, Alnouri G, Sataloff RT (2020). Otolaryngology-related Google Search trends during the COVID-19 pandemic. Am J Otolaryngol.

[3] Liu DT, Besser G, Leonhard M, Bartosik TJ, Parzefall T, Brkic FF, Mueller CA, Riss D (2020). Seasonal variations in public inquiries into laryngitis: an infodemiology study. J Voice.

[4] Liu DT, Besser G, Parzefall T, Riss D, Mueller CA (2020). Winter peaks in web-based public inquiry into epistaxis. Eur Arch Otorhinolaryngol.

[5] Plante DT, Ingram DG (2015). Seasonal trends in tinnitus symptomatology: evidence from Internet search engine query data. Eur Arch Otorhinolaryngol.

[6] Faoury M, Upile T, Patel N (2019). Using Google Trends to understand information-seeking behaviour about throat cancer. J. Laryngol. Otol.

[7] Trohman RG, Sharma PS, McAninch EA, Bianco AC (2019). Amiodarone and thyroid physiology, pathophysiology, diagnosis and management. Trends Cardiovasc Med.

[8] Zuin M, Rigatelli G, Ronco F (2020). Worldwide and European interest in the MitraClip. Journal of Cardiovascular Medicine.

[9] Dreher PC, Tong C, Ghiraldi E, Friedlander JI (2018). Use of Google Trends to track online behavior and interest in kidney stone surgery. Urology.

[10] Ikpeze TC, Mesfin A (2018). Interest in orthopedic surgery residency: a Google Trends analysis. J Surg Orthop Adv.

[11] Skopelja EN, Whipple EC, Richwine P (2008). Reaching and teaching teens: adolescent health literacy and the internet. Journal of Consumer Health On the Internet.

[12] Harmes KM, Blackwood RA, Burrows HL, Cooke JM, Harrison RV, Passamani PP (2013). Otitis media: diagnosis and treatment. Am Fam Physician.

[13] Morley G (1938). Otitis externa. Br Med J.

[14] Lieberthal AS, Carroll AE, Chonmaitree T, Ganiats Theodore G, Hoberman Alejandro, Jackson Mary Anne, Joffe Mark D, Miller Donald T, Rosenfeld Richard M, Sevilla Xavier D, Schwartz Richard H, Thomas Pauline A, Tunkel David E (2013). The diagnosis and management of acute otitis media. Pediatrics.

[15] Schaefer P, Baugh R (2012). Acute otitis externa: an update. Am Fam Physician.

[16] Brkic FF, Besser G, Janik S, Gadenstaetter AJ, Parzefall T, Riss D, Liu DT (2021). Peaks in online inquiries into pharyngitis-related symptoms correspond with annual incidence rates. Eur Arch Otorhinolaryngol.

[17] Knopke S, Böttcher A, Chadha P, Olze H, Bast F (2017). [Seasonal differences of tympanogram and middle ear findings in children] German version. HNO.

[18] Castagno LA, Lavinsky L (2002). Otitis media in children: seasonal changes and socioeconomic level. International Journal of Pediatric Otorhinolaryngology.

[19] Gordon MA, Grunstein E, Burton WB (2004). The effect of the season on otitis media with effusion resolution rates in the New York Metropolitan area. Int J Pediatr Otorhinolaryngol.

[20] Gharaghani M, Seifi Z, Zarei Mahmoudabadi A (2015). Otomycosis in Iran: a review. Mycopathologia.

[21] Centers for Disease ControlPrevention (CDC) (2011). Estimated burden of acute otitis externa--United States, 2003-2007. MMWR Morb Mortal Wkly Rep.

[22] Lu YX, Liang JQ, Gu QL, Yu XM, Yan X (2017). [Study on the correlation between meteorological factors and acute otitis media in outpatients of children in Beijing]. Zhonghua Er Bi Yan Hou Tou Jing Wai Ke Za Zhi.

[23] Crowson M, Schulz K, Tucci D (2016). National utilization and forecasting of ototopical antibiotics: Medicaid data versus "Dr. Google". Otology Neurotology.

[24] FAQ about Google Trends data. Google.

[25] Adrian GB, Annette JD (2009). Analysing Seasonal Health Data.

[26] van Rossum G (1995). Python tutorial. Centrum Wiskunde & Informatica Stichting.

[27] Harris CR, Millman KJ, van der Walt SJ, Gommers R, Virtanen P, Cournapeau D, Wieser E, Taylor J, Berg S, Smith NJ, Kern R, Picus M, Hoyer S, van Kerkwijk MH, Brett M, Haldane A, Del Río Jaime Fernández, Wiebe M, Peterson P, Gérard-Marchant Pierre, Sheppard K, Reddy T, Weckesser W, Abbasi H, Gohlke C, Oliphant TE (2020). Array programming with NumPy. Nature.

[28] The Pandas development team. Pandas.

[29] Hunter JD (2007). Matplotlib: a 2D graphics environment. Comput. Sci. Eng.

[30] Jordahl K GeoPandas: Python tools for geographic data. GeoPandas 0.9.0.

[31] Patthi B (2017). Global search trends of oral problems using Google Trends from 2004 to 2016: an exploratory analysis. JCDR.

[32] Shen JK, Every J, Morrison SD, Massenburg BB, Egbert MA, Susarla SM (2020). Global interest in oral and maxillofacial surgery: analysis of Google Trends data. J Oral Maxillofac Surg.

[33] Harorli OT, Harorli H (2014). Evaluation of internet search trends of some common oral problems, 2004 to 2014. Community Dent Health.

[34] Samaras L, García-Barriocanal Elena, Sicilia M (2017). Syndromic surveillance models using web data: the case of influenza in Greece and Italy using Google Trends. JMIR Public Health Surveill.

[35] Zhang Y, Bambrick H, Mengersen K, Tong S, Hu W (2018). Using Google Trends and ambient temperature to predict seasonal influenza outbreaks. Environ Int.

[36] Schnoell J, Besser G, Jank BJ, Bartosik TJ, Parzefall T, Riss D, Mueller CA, Liu DT (2021). The association between COVID-19 cases and deaths and web-based public inquiries. Infect Dis (Lond).

[37] Walker A, Hopkins C, Surda P (2020). Use of Google Trends to investigate loss-of-smell-related searches during the COVID-19 outbreak. Int Forum Allergy Rhinol.

[38] Venkatesh U, Gandhi PA (2020). Prediction of COVID-19 outbreaks using Google Trends in India: a retrospective analysis. Healthc Inform Res.

[39] Ayyoubzadeh S, Ayyoubzadeh S, Zahedi H, Ahmadi M, R Niakan Kalhori Sharareh (2020). Predicting COVID-19 incidence through analysis of Google Trends data in Iran: data mining and deep learning pilot study. JMIR Public Health Surveill.

[40] Marchetti F, Ronfani L, Nibali SC, Tamburlini G, Italian Study Group on Acute Otitis Media (2005). Delayed prescription may reduce the use of antibiotics for acute otitis media: a prospective observational study in primary care. Arch Pediatr Adolesc Med.

[41] Jacobs MR, Dagan R, Appelbaum PC, Burch DJ (1998). Prevalence of antimicrobial-resistant pathogens in middle ear fluid: multinational study of 917 children with acute otitis media. Antimicrob Agents Chemother.

[42] Arrieta A, Singh J (2004). Management of recurrent and persistent acute otitis media: new options with familiar antibiotics. Pediatr Infect Dis J.

[43] Kaushik V, Malik T, Saeed S (2010). Interventions for acute otitis externa. Cochrane Database Syst Rev.

[44] Roland PS, Stroman DW (2002). Microbiology of acute otitis externa. Laryngoscope.

[45] Laulajainen-Hongisto A, Aarnisalo AA, Jero J (2016). Differentiating acute otitis media and acute mastoiditis in hospitalized children. Curr Allergy Asthma Rep.

[46] van den Aardweg Maaike T A, Rovers M, de Ru J Alexander, Albers F, Schilder A (2008). A systematic review of diagnostic criteria for acute mastoiditis in children. Otol Neurotol.

[47] Stalfors J, Enoksson F, Hermansson A, Hultcrantz M, Stenfeldt K, Groth A, Robinson (2013). National assessment of validity of coding of acute mastoiditis: a standardised reassessment of 1966 records. Clin Otolaryngol.

[48] Psarommatis IM, Voudouris C, Douros K, Giannakopoulos P, Bairamis T, Carabinos C (2012). Algorithmic management of pediatric acute mastoiditis. Int J Pediatr Otorhinolaryngol.

[49] Quesnel S, Nguyen M, Pierrot S, Contencin P, Manach Y, Couloigner V (2010). Acute mastoiditis in children: a retrospective study of 188 patients. Int J Pediatr Otorhinolaryngol.

[50] Amaro CE, Espiney R, Radu L, Guerreiro F (2019). Malignant (necrotizing) externa otitis: the experience of a single hyperbaric centre. Eur Arch Otorhinolaryngol.

[51] Babiatzki A, Sadé J (1987). Malignant external otitis. J Laryngol Otol.

[52] Pichichero M E (2000). Recurrent and persistent otitis media. Pediatr Infect Dis J.

[53] Bhattacharyya N, Kepnes LJ (2011). Initial impact of the acute otitis externa clinical practice guideline on clinical care. Otolaryngol Head Neck Surg.

[54] Walker A, Hopkins C, Surda P (2020). Use of Google Trends to investigate loss-of-smell-related searches during the COVID-19 outbreak. Int Forum Allergy Rhinol.

[55] Telfer S, Woodburn J (2015). Let me Google that for you: a time series analysis of seasonality in internet search trends for terms related to foot and ankle pain. J Foot Ankle Res.

