# Supplementary material for: Biannual Differences in Interest Peaks for Web Inquiries Into Ear Pain and Ear Drops: Infodemiology Study
Source: J Med Internet Res. 2021 Jun 24;23(6):e28328. doi: 10.2196/28328 (PMC8277359; doi:10.2196/28328)
Supplement: Multimedia Appendix 1 [file jmir_v23i6e28328_app1.docx]

**Supplementary Table 1**. Results from relative search volume comparison between primary and related search terms in Australia.

| **Primary search term** | **Mean relative search volume** | **Related search term** | **Mean relative search volume** |
| --- | --- | --- | --- |
| Ear drops | 16.6 | Ear infection | 59.3 |
| Ear drops | 24.0 | Blocked ear | 35.9 |
| Ear drops | 41.9 | Ear wax drops | 5.6 |
|  |  | Otodex | 9.3 |
|  |  | Otodex ear drops | 4.8 |
|  |  | Sofradex ear drops | 4.3 |
|  |  | Waxsol ear drops | 3.3 |
|  |  | Sofradex | 11.1 |
|  |  | Waxsol | 9.8 |
|  |  | Ear drops for infection | 2.7 |
|  |  | Ear drops for ear infection | 2.3 |
|  |  | Ciproxin | 12.8 |
|  |  | Ciproxin ear drops | 3.7 |
|  |  | Otocomb | 9.4 |
|  |  | Ear drops chemist warehouse | 1.6 |
|  |  | Kenacomb ear drops | 2.5 |
|  |  | Swimmers ear | 17.3 |
|  |  | Ciloxan | 5.2 |
|  |  | Swimmers ear drops | 2.2 |
|  |  | Kenacomb | 17.9 |
|  |  | Otocomb ear drops | 1.9 |
|  |  | Ear wax removal | 21.4 |
|  |  | Ciloxan ear drops | 2.2 |
|  |  | Ear clear drops | 2.1 |
|  |  | Antibiotic ear drops | 1.4 |
| Ear infection | 57.2 | Ear infections | 13.6 |
| Ear infection | 57.2 | Blocked ear | 23.1 |
| Ear infection | 57.2 | Ear drops for ear infection | 0.8 |
| Ear infection | 59.3 | Middle ear infection | 11.0 |
|  |  | Ear pain | 30.2 |
|  |  | Inner ear infection | 6.3 |
|  |  | Inner ear | 19.2 |
|  |  | Ear infection treatment | 3.9 |
|  |  | Symptoms of ear infection | 3.7 |
|  |  | Ear infection in adults | 3.1 |
|  |  | Sinus infection | 39.5 |
|  |  | Throat infection | 21.2 |
|  |  | Ear drops | 16.6 |
|  |  | Middle ear infection symptoms | 2.43 |
|  |  | Baby ear infection | 2.0 |
|  |  | Outer ear infection | 1.8 |
|  |  | Ear infection signs | 1.6 |
|  |  | Fungal ear infection | 1.6 |
|  |  | Antibiotics for ear infection | 1.7 |
|  |  | Signs of ear infection | 1.4 |
|  |  | Ear infection antibiotic | 1.3 |
|  |  | Earache | 14.2 |
|  |  | Middle ear infection adults | 1.7 |
|  |  | Viral ear infection | 1.6 |
|  |  | Ear infection symptoms adults | 1.5 |
| Ear pain | 24.0 | Neck pain | 57.2 |
| Ear pain | 30.2 | Ear infection | 59.3 |
| Ear pain | 41.9 | Pain in ear | 14.4 |
| Ear pain | 44.2 | Sore ear | 20.8 |
|  |  | Blocked ear | 35.8 |
| Ear pain | 45.2 | Earache | 21.0 |
|  |  | Pain behind ear | 5.8 |
|  |  | Jaw pain | 22.3 |
|  |  | Ear pain causes | 3.2 |
|  |  | Sharp pain in ear | 2.6 |
|  |  | Ear pain relief | 1.9 |
|  |  | Ear infection symptoms | 15.5 |
|  |  | Inner ear pain | 1.6 |
|  |  | Sore throat and ear pain | 1.4 |
|  |  | Ear pain cold | 1.6 |
|  |  | Middle ear infection | 16.5 |
|  |  | Jaw pain near ear | 1.2 |
| Otitis | 23.0 | Ear infection | 59.3 |
| Otitis | 34.0 | Eustachian tube | 18.0 |
| Otitis | 34.2 | Acute otitis media | 2.9 |
|  |  | Suppurative otitis media | 1.5 |
| Otitis | 39.2 | Otitis media | 28.0 |
|  |  | Otitis externa | 11.1 |
|  |  | Chronic otitis media | 3.8 |
|  |  | Otitis media with effusion | 2.4 |
|  |  | Otitis media rch | 1.1 |
|  |  | Serous otitis media | 1.8 |
|  |  | Chronic suppurative otitis media | 1.4 |
|  |  | Malignant otitis externa | 1.3 |
|  |  | Glue ear | 16.2 |
|  |  | Middle ear infection | 18.8 |
|  |  | Sofradex | 7.5 |
|  |  | Ome | 3.3 |
|  |  | Eustachian tube dysfunction | 7.4 |
| Otitis media | 15.3 | Ear infection | 56.4 |
| Otitis media | 21.3 | Acute otitis media | 3.2 |
|  |  | Chronic otitis media | 2.1 |
|  |  | Otitis media with effusion | 1.3 |
|  |  | Otitis externa | 9.3 |
|  |  | Suppurative otitis media | 1.5 |
|  |  | Otitis media rch | 0.7 |
|  |  | Serous otitis media | 1.4 |
|  |  | Chronic suppurative otitis media | 1.0 |
|  |  | Middle ear infection | 15.7 |
|  |  | Ome | 2.6 |
|  |  | Mastoiditis | 5.9 |

**Supplementary Table 2**. Results from relative search volume comparison between primary and related search terms in Canada.

| **Primary search term** | **Mean relative search volume** | **Related search term** | **Mean relative search volume** |
| --- | --- | --- | --- |
| Ear drops | 11.9 | Ear infection | 59.7 |
| Ear drops | 18.0 | Polysporin | 47.2 |
| Ear drops | 26.9 | Ciprofloxacin | 68.1 |
| Ear drops | 40.1 | Earache | 44.5 |
| Ear drops | 36.6 | Ciprodex | 18.4 |
|  |  | Surolan | 6.9 |
|  |  | Ear drops for pain | 1.2 |
| Ear drops | 40.1 | Polysporin ear drops | 6.4 |
|  |  | Ear infection drops | 6.4 |
|  |  | Ear wax drops | 4.8 |
|  |  | Ear drops for ear infection | 3.3 |
|  |  | Ciprodex ear drops | 3.3 |
|  |  | Murine ear drops | 2.7 |
|  |  | Over the counter ear drops | 2.3 |
|  |  | Polysporin eye drops | 16.0 |
|  |  | Swimmers ear | 20.7 |
|  |  | Swimmers ear drops | 2.2 |
|  |  | Cerumol | 6.4 |
|  |  | Ear wax removal | 33.4 |
|  |  | Cerumol ear drops | 2.1 |
|  |  | Antibiotic ear drops | 2.3 |
|  |  | Auralgan | 6.5 |
|  |  | Auralgan ear drops | 1.9 |
|  |  | Surolan ear drops | 1.8 |
|  |  | Locacorten vioform | 5.5 |
| Ear infection | 48.2 | Sinus infection | 49.3 |
| Ear infection | 57.9 | Baby ear infection | 1.4 |
| Ear infection | 59.2 | Inner ear | 20.2 |
|  |  | Inner ear infection | 6.5 |
|  |  | Ear pain | 33.8 |
|  |  | Ear infection adults | 4.5 |
|  |  | Symptoms of ear infection | 4.3 |
|  |  | Ear infection in adults | 3.1 |
|  |  | Ear infections | 15.1 |
|  |  | Ear infection treatment | 3.2 |
|  |  | Throat infection | 19.2 |
|  |  | Ear infection signs | 2.4 |
|  |  | Middle ear infection | 3.0 |
|  |  | Signs of ear infection | 2.4 |
|  |  | Ear infection drops | 1.9 |
|  |  | Ear drops | 12.0 |
|  |  | Ear infection symptoms adults | 1.8 |
|  |  | Pain in ear | 11.3 |
|  |  | Inner ear infection symptoms | 2.1 |
|  |  | Ear infection remedies | 1.9 |
|  |  | Adult ear infection | 2.8 |
|  |  | Ear infection causes | 1.8 |
|  |  | Ear infection symptoms in adults | 1.4 |
|  |  | Ear infection remedy | 1.9 |
|  |  | Antibiotics for ear infection | 1.1 |
| Ear pain | 27.2 | Neck pain | 57.4 |
| Ear pain | 27.4 | Sinus infection | 49.3 |
| Ear pain | 33.8 | Ear infection | 59.2 |
| Ear pain | 50.7 | Pain in ear | 16.9 |
|  |  | Pain behind ear | 5.9 |
|  |  | Jaw pain | 24.4 |
|  |  | Left ear pain | 4.1 |
|  |  | Ear and throat pain | 3.0 |
|  |  | Sore throat ear pain | 2.1 |
|  |  | Ear and jaw pain | 2.9 |
|  |  | Ear pressure pain | 2.9 |
|  |  | Ear pain causes | 2.5 |
|  |  | Sharp pain in ear | 2.5 |
|  |  | Inner ear pain | 2.6 |
|  |  | Earache pain | 3.2 |
|  |  | Ear ache | 13.9 |
|  |  | Earache | 19.8 |
|  |  | Ear infection symptoms | 18.3 |
|  |  | Ear pain relief | 1.9 |
|  |  | Ear pain in adults | 1.8 |
|  |  | Pain in ear and jaw | 1.3 |
|  |  | Ear drops | 18.0 |
|  |  | Ear pain remedies | 1.2 |
| Otite | 26.6 | Oreille | 49.3 |
| Otite | 40.6 | Sinusite | 43.5 |
| Otite | 46.6 | Otite adulte | 5.3 |
|  |  | Otite chien | 3.0 |
|  |  | Otite symptome | 2.9 |
|  |  | Otite externe | 3.1 |
|  |  | Otite enfant | 2.4 |
|  |  | Symptomes otite | 2.5 |
|  |  | Otite séreuse | 2.0 |
|  |  | Otite baigneur | 1.9 |
|  |  | Otite bebe | 1.5 |
|  |  | Soulager otite | 1.3 |
|  |  | Iness otite | 1.1 |
|  |  | Antibiotique | 32.9 |
|  |  | Oreille bouchée | 9.2 |
| Otite | 48.2 | Otite interne | 1.7 |
|  |  | Otite du baigneur | 1.9 |
|  |  | Otite sereuse | 1.7 |
|  |  | Otite inesss | 0.9 |
|  |  | Otite symptômes | 1.3 |
|  |  | Otite contagieux | 0.8 |
|  |  | Symptome otite adulte | 0.9 |
|  |  | Otite contagion | 0.8 |
|  |  | Otite séreuse adulte | 0.6 |
| Otitis | 13.8 | Ear infection | 59.2 |
| Otitis | 28.9 | Otite | 48.4 |
| Otitis | 38.0 | Otitis media | 23.7 |
|  |  | Otitis externa | 9.7 |
|  |  | Acute otitis media | 4.2 |
|  |  | Serous otitis media | 2.9 |
|  |  | Otitis media with effusion | 3.0 |
|  |  | Otitis media treatment | 3.2 |
|  |  | Cps otitis media | 0.9 |
|  |  | What is otitis media | 0.9 |
|  |  | Malignant otitis externa | 1.4 |
|  |  | Swimmers ear | 17.2 |
|  |  | Ome | 3.8 |
|  |  | Otite en anglais | 0.7 |
| Otitis | 49.0 | Otitis media symptoms | 2.3 |
| Otitis media | 8.6 | Ear infection | 59.2 |
| Otitis media | 28.4 | Otitis media with effusion | 3.0 |
| Otitis media | 31.4 | Otitis media treatment | 4.1 |
|  |  | Otitis media symptoms | 2.2 |
| Otitis media | 32.9 | acute otitis media | 5.8 |
|  |  | serous otitis media | 4.3 |
|  |  | otitis externa | 12.8 |
|  |  | what is otitis media | 1.5 |
|  |  | ome | 5.4 |

**Supplementary Table 3**. Results from relative search volume comparison between primary and related search terms in UK.

| **Primary search term** | **Mean relative search volume** | **Related search term** | **Mean relative search volume** |
| --- | --- | --- | --- |
| Ear drops | 13.7 | Ear infection | 41.5 |
| Ear drops | 17.8 | Ear wax | 40.4 |
| Ear drops | 19.4 | Ear pain | 37.1 |
| Ear drops | 40.3 | Blocked ear | 43.7 |
| Ear drops | 40.3 | Otex | 10.9 |
|  |  | Otex ear drops | 5.8 |
|  |  | Ear infection drops | 4.1 |
|  |  | Olive oil ear drops | 3.2 |
|  |  | Olive oil | 39.0 |
|  |  | Bicarbonate ear drops | 3.2 |
|  |  | Sodium bicarbonate | 13.4 |
|  |  | Ear drops boots | 2.6 |
|  |  | Ear drops for ear infection | 2.0 |
|  |  | Antibiotic ear drops | 1.9 |
|  |  | Ear pain drops | 1.7 |
|  |  | Best ear drops | 1.4 |
|  |  | Over the counter ear drops | 1.1 |
|  |  | Ear wax removal drops | 1.3 |
|  |  | Ear wax removal | 25.7 |
|  |  | Canaural | 3.4 |
|  |  | Canaural ear drops | 1.6 |
|  |  | Sofradex ear drops | 1.4 |
|  |  | Sofradex | 4.3 |
|  |  | Surolan | 2.5 |
|  |  | Ear drops for dogs | 1.1 |
| Ear infection | 41.6 | Ear infection symptoms | 7.3 |
|  |  | Ear pain | 26.2 |
|  |  | Inner ear infection | 3.8 |
|  |  | Ear infection pain | 3.4 |
|  |  | Ear infection adults | 3.0 |
|  |  | Ear infection in adults | 2.3 |
|  |  | Symptoms of ear infection | 2.2 |
|  |  | Middle ear infection | 2.4 |
|  |  | Ear infection antibiotics | 2.1 |
|  |  | Ear infection nhs | 1.9 |
|  |  | Throat infection | 15.9 |
|  |  | Ear infection treatment | 1.6 |
|  |  | Ear infection drops | 1.4 |
|  |  | Ear infections | 7.6 |
|  |  | Ear drops | 13.7 |
|  |  | Pain in ear | 10.2 |
|  |  | Antibiotics for ear infection | 1.1 |
|  |  | Sinus infection | 19.1 |
|  |  | Ear infection signs | 1.0 |
|  |  | Sore ear | 7.6 |
|  |  | Blocked ear | 14.9 |
|  |  | Signs of ear infection | 1.0 |
|  |  | Earache | 17.7 |
|  |  | Outer ear infection | 1.1 |
|  |  | Throat and ear infection | 0.9 |
| Ear pain | 20.8 | Neck pain | 46.8 |
| Ear pain | 26.1 | Ear infection | 41.5 |
| Ear pain | 37.1 | Head pain | 45.0 |
| Ear pain | 37.1 | Pain in ear | 14.5 |
|  |  | Ear infection pain | 4.8 |
|  |  | Pain behind ear | 4.6 |
|  |  | Left ear pain | 2.8 |
|  |  | Jaw pain | 15.5 |
|  |  | Ear and neck pain | 2.5 |
|  |  | Right ear pain | 2.5 |
|  |  | Pain in neck | 30.0 |
|  |  | Sore ear | 10.8 |
|  |  | Throat and ear pain | 2.2 |
|  |  | Sharp ear pain | 2.1 |
|  |  | Pain in the ear | 2.1 |
|  |  | Jaw and ear pain | 1.7 |
|  |  | Pain in left ear | 1.6 |
|  |  | Sharp pain in ear | 1.4 |
|  |  | Pain in neck and ear | 1.5 |
|  |  | Earache | 25.0 |
|  |  | Pain in my ear | 1.4 |
|  |  | Ear ache | 12.2 |
|  |  | Blocked ear pain | 1.3 |
|  |  | Blocked ear | 21.1 |
|  |  | Pain in right ear | 1.3 |
| Otitis | 10.9 | Ear infection | 41.5 |
| Otitis | 15.5 | Ear pain | 37.1 |
| Otitis | 36.0 | Effusion | 40.8 |
| Otitis | 32.8 | Otitis externa | 12.5 |
|  |  | Malignant otitis externa | 0.9 |
|  |  | Cks otitis externa | 0.5 |
|  |  | Mastoiditis | 6.6 |
|  |  | Chronic suppurative otitis media | 0.5 |
| Otitis | 36.0 | Otitis media | 19.4 |
|  |  | Acute otitis media | 2.5 |
|  |  | Otitis media with effusion | 2.2 |
|  |  | Otitis externa treatment | 1.8 |
|  |  | Chronic otitis media | 2.5 |
|  |  | Otitis media treatment | 1.9 |
|  |  | Otitis media in adults | 1.2 |
|  |  | Suppurative otitis media | 1.2 |
|  |  | Cks otitis media | 0.7 |
|  |  | Otitis media symptoms | 1.2 |
|  |  | Glue ear | 27.7 |
|  |  | Otitis externa symptoms | 0.9 |
|  |  | Otomize | 10.6 |
|  |  | Serous otitis media | 1.1 |
|  |  | Ear infection symptoms | 24.0 |
|  |  | Chronic otitis externa | 1.1 |
|  |  | What is otitis media | 0.8 |
| Otitis media | 5.9 | Ear infection | 42.6 |
| Otitis media | 12.7 | Earache | 39.6 |
| Otitis media | 23.9 | Ear infections | 32.3 |
| Otitis media | 28.2 | Glue ear | 43.5 |
| Otitis media | 28.2 | Ear infection symptoms | 35.4 |
| Otitis media | 28.2 | Acute otitis media | 3.4 |
|  |  | Otitis externa | 20.5 |
|  |  | Otitis media with effusion | 2.6 |
|  |  | Otitis media treatment | 2.4 |
|  |  | Suppurative otitis media | 1.6 |
|  |  | Otitis media cks | 0.9 |
|  |  | Serous otitis media | 1.0 |
|  |  | Tympanic membrane | 8.6 |
|  |  | Otitis media nice cks | 0.3 |
|  |  | Ome | 4.4 |
| Otitis media | 34.0 | Chronic otitis media | 3.7 |
|  |  | Otitis media symptoms | 2.4 |
|  |  | Otitis media in adults | 2.6 |
|  |  | What is otitis media | 1.1 |
|  |  | Chronic suppurative otitis media | 1.3 |
| Otitis media | 42.4 | Otitis media patient uk | 0.7 |

**Supplementary Table 4**. Results from relative search volume comparison between primary and related search terms in USA.

| **Primary search term** | **Mean relative search volume** | **Related search term** | **Mean relative search volume** |
| --- | --- | --- | --- |
| Ear drops | 10.6 | Ear infection | 55.9 |
| Ear drops | 10.8 | Eye drops | 47.2 |
| Ear drops | 16.4 | Ear pain | 50.2 |
| Ear drops | 19.1 | Ear wax | 54.8 |
| Ear drops | 31.0 | Swimmers ear | 23.9 |
| Ear drops | 34.1 | Ear infection ear drops | 4.9 |
|  |  | Ear infection drops | 4.9 |
|  |  | Ear drops swimmers ear | 3.1 |
|  |  | Swimmers ear drops | 3.2 |
|  |  | Ear drops for ear infection | 2.8 |
|  |  | Ear pain drops | 2.8 |
|  |  | Ear wax drops | 3.0 |
|  |  | Ear drops for ear pain | 1.7 |
|  |  | Drops for ear pain | 1.6 |
|  |  | Ear drops over the counter | 1.8 |
|  |  | Ear antibiotic drops | 1.5 |
|  |  | Otic drops | 4.5 |
|  |  | Otic ear drops | 1.7 |
|  |  | Ofloxacin drops | 3.4 |
|  |  | Ofloxacin | 12.2 |
|  |  | Debrox drops | 1.7 |
|  |  | Debrox | 9.2 |
|  |  | Ofloxacin ear drops | 1.2 |
|  |  | Debrox ear drops | 1.2 |
|  |  | Neomycin | 10.6 |
| Ear infection | 40.1 | Sinus infection | 51.5 |
| Ear infection | 55.9 | Symptoms ear infection | 9.5 |
|  |  | Ear infection pain | 4.5 |
|  |  | Ear pain | 32.2 |
|  |  | Ear infection adults | 3.6 |
|  |  | Inner ear | 16.1 |
|  |  | Inner ear infection | 4.4 |
|  |  | Sinus ear infection | 3.3 |
|  |  | Symptoms of ear infection | 3.0 |
|  |  | Ear infection in adults | 2.6 |
|  |  | Ear infection signs | 2.2 |
|  |  | Ear infections | 13.7 |
|  |  | Ear infection antibiotics | 2.1 |
|  |  | Ear infection treatment | 2.4 |
|  |  | Middle ear infection | 2.2 |
|  |  | Throat infection | 11.9 |
|  |  | Signs of ear infection | 1.7 |
|  |  | Ear infection drops | 1.5 |
|  |  | Ear infection remedies | 1.6 |
|  |  | Ear drops | 10.6 |
|  |  | Ear infection adult | 2.2 |
|  |  | Treat ear infection | 1.3 |
|  |  | Antibiotics for ear infection | 1.1 |
|  |  | Ear infection symptoms adults | 1.3 |
|  |  | Ear infection causes | 1.3 |
| Ear pain | 16.5 | Sore throat | 50.8 |
| Ear pain | 30.6 | Neck pain | 62.1 |
| Ear pain | 32.5 | Ear infection | 55.9 |
| Ear pain | 50.2 | Pain in ear | 13.6 |
|  |  | Ear infection pain | 6.9 |
|  |  | Pain behind ear | 4.6 |
|  |  | Ear throat pain | 4.7 |
|  |  | Jaw pain | 21.4 |
|  |  | Ear jaw pain | 3.4 |
|  |  | Left ear pain | 3.2 |
|  |  | Right ear pain | 3.0 |
|  |  | Ear and throat pain | 2.8 |
|  |  | Sharp ear pain | 2.8 |
|  |  | Ear pain cause | 2.5 |
|  |  | Neck and ear pain | 2.5 |
|  |  | Sore throat ear pain | 2.2 |
|  |  | Ear pressure pain | 2.2 |
|  |  | Pain in the ear | 2.3 |
|  |  | Ear pressure | 13.1 |
|  |  | Headache ear pain | 2.0 |
|  |  | Sinus ear pain | 2.2 |
|  |  | Jaw and ear pain | 2.0 |
|  |  | Ear pain causes | 1.8 |
|  |  | Inner ear pain | 1.8 |
|  |  | Sharp pain in ear | 1.6 |
| Otitis | 14.7 | Ear infection | 55.9 |
| Otitis | 35.7 | Sinusitis | 65.6 |
| Otitis | 42.0 | Effusion | 68.3 |
| Otitis | 67.8 | Otitis media | 44.4 |
|  |  | Otitis externa | 14.0 |
|  |  | Otitis media treatment | 4.2 |
|  |  | Otitis media effusion | 3.2 |
|  |  | Chronic otitis | 3.3 |
|  |  | Otitis media with effusion | 2.7 |
|  |  | What is otitis | 2.1 |
|  |  | Chronic otitis media | 2.6 |
|  |  | Suppurative | 7.2 |
|  |  | What is otitis media | 1.5 |
|  |  | Otitis external | 2.1 |
|  |  | Otitis media in adults | 1.8 |
|  |  | Icd 10 for otitis media | 0.8 |
|  |  | Icd 10 code otitis media | 0.9 |
|  |  | Otitis media icd 9 | 1.1 |
| Otitis | 69.3 | Acute otitis | 6.7 |
|  |  | Otitis media icd 10 | 2.1 |
|  |  | Otitis media symptoms | 1.8 |
|  |  | Right otitis media | 0.9 |
|  |  | Left otitis media | 0.8 |
|  |  | Icd 10 otitis externa | 0.7 |
|  |  | Icd 10 code for otitis media | 0.7 |
| Otitis media | 9.3 | Ear infection | 55.1 |
| Otitis media | 26.9 | Effusion | 68.2 |
| Otitis media | 58.6 | Acute otitis media | 7.7 |
|  |  | Icd 10 otitis media | 2.8 |
|  |  | Serous otitis | 6.1 |
|  |  | Serous otitis media | 4.8 |
|  |  | Otitis media effusion | 4.2 |
|  |  | Otitis media with effusion | 3.5 |
|  |  | Otitis externa | 18.7 |
|  |  | Icd 10 for otitis media | 1.1 |
|  |  | Otitis media in adults | 2.0 |
|  |  | Right otitis media | 1.2 |
|  |  | Left otitis media | 1.3 |
|  |  | Icd 10 code for otitis media | 0.9 |
|  |  | Otitis media icd 9 | 1.4 |
|  |  | Bilateral otitis media | 1.3 |
|  |  | Treatment of otitis media | 1.6 |
|  |  | Left otitis media icd 10 | 0.6 |
|  |  | Right otitis media icd 10 | 0.6 |
| Otitis media | 72.3 | Chronic otitis media | 4.0 |
|  |  | Otitis media symptoms | 3.1 |
|  |  | Suppurative | 11.9 |
|  |  | What is otitis media | 2.5 |
|  |  | Icd 10 code otitis media | 1.5 |
|  |  | Treatment for otitis media | 2.2 |

**Supplementary Table 5**. Results from relative search volume comparison between primary and related search terms in Germany.

| **Primary search term** | **Mean relative search volume** | **Related search term** | **Mean relative search volume** |
| --- | --- | --- | --- |
| Gehörgangsentzündung | 5.3 | Mittelohrentzündung | 43.0 |
| Gehörgangsentzündung | 12.1 | Gehörschutz | 54.1 |
| Gehörgangsentzündung | 17.6 | Ohrentropfen | 39.8 |
| Gehörgangsentzündung | 23.0 | Ohrenschmerzen | 39.1 |
| Gehörgangsentzündung | 25.7 | Gehörgang | 32.3 |
| Gehörgangsentzündung | 35.5 | Gehörgangsentzündung dauer | 2.2 |
|  |  | Gehörgangsentzündung hausmittel | 2.0 |
|  |  | Gehörgangsentzündung symptome | 1.8 |
|  |  | Gehörgangsentzündung schmerzen | 1.7 |
|  |  | Gehörgangsentzündung ansteckend | 0.7 |
|  |  | Gehörgangsentzündung behandlung | 1.1 |
|  |  | Otalgan | 23.0 |
|  |  | Gehörgangentzündung | 7.2 |
|  |  | Gehörgangsentzündung kühlen | 0.4 |
| Mittelohrentzündung | 10.8 | Ohr | 53.8 |
| Mittelohrentzündung | 24.3 | Ohrenschmerzen | 39.1 |
| Mittelohrentzündung | 39.2 | Hörsturz | 52.1 |
| Mittelohrentzündung | 43.0 | Symptome mittelohrentzündung | 4.5 |
|  |  | Hausmittel mittelohrentzündung | 2.4 |
|  |  | Baby mittelohrentzündung | 1.7 |
|  |  | Mittelohrentzündung schmerzen | 1.2 |
|  |  | Mittelohrentzündung kinder | 1.1 |
|  |  | Antibiotika mittelohrentzündung | 0.9 |
|  |  | Mittelohrentzündung bei kindern | 1.0 |
|  |  | Mittelohrentzündung kleinkind | 0.9 |
|  |  | Mittelohrentzündung was tun | 0.6 |
|  |  | Mittelohrentzündung kind | 0.8 |
|  |  | Behandlung mittelohrentzündung | 0.9 |
|  |  | Mittelohrentzündung fliegen | 0.8 |
|  |  | Trommelfell | 15.8 |
|  |  | Zwiebel mittelohrentzündung | 0.6 |
|  |  | Chronische mittelohrentzündung | 0.7 |
|  |  | Mittelohrentzündung ansteckend | 0.5 |
|  |  | Mittelohrentzündung tinnitus | 0.6 |
|  |  | Mittelohrentzündung homöopathie | 0.8 |
|  |  | Zwiebelsäckchen | 3.6 |
|  |  | Mittelohrentzündung zwiebelsäckchen | 0.5 |
|  |  | Mittelohrentzündung schwindel | 0.5 |
|  |  | Ohrentropfen | 11.9 |
| Ohr entzündung | 3.1 | Mittelohrentzündung | 43.0 |
| Ohr entzündung | 23.4 | Entzündung im ohr | 9.3 |
|  |  | Entzündung am ohr | 2.6 |
|  |  | Entzündung hinterm ohr | 0.8 |
| Ohrenschmerzen | 12.7 | Hausmittel | 46.3 |
| Ohrenschmerzen | 17.3 | Ohr | 53.3 |
| Ohrenschmerzen | 40.4 | Ohrenschmerzen hausmittel | 4.4 |
|  |  | Gegen ohrenschmerzen | 2.7 |
|  |  | Erkältung ohrenschmerzen | 1.8 |
|  |  | Ohrenschmerzen halsschmerzen | 1.7 |
|  |  | Ohrenschmerzen kind | 1.4 |
|  |  | Ohrenschmerzen was tun | 1.5 |
|  |  | Zwiebel ohrenschmerzen | 1.4 |
|  |  | Kopfschmerzen ohrenschmerzen | 1.3 |
|  |  | Ohrenschmerzen kinder | 1.2 |
|  |  | Ohrenschmerzen bei kindern | 1.2 |
|  |  | Hals ohrenschmerzen | 1.2 |
|  |  | Mittelohrentzündung | 24.9 |
|  |  | Ohrenschmerzen einseitig | 0.8 |
|  |  | Hausmittel bei ohrenschmerzen | 0.8 |
|  |  | Ohrenschmerzen druck | 0.9 |
|  |  | Hals und ohrenschmerzen | 0.7 |
|  |  | Starke ohrenschmerzen | 0.8 |
|  |  | Ohrenschmerzen zwiebelsäckchen | 0.7 |
|  |  | Hausmittel gegen ohrenschmerzen | 0.7 |
|  |  | Homöopathie ohrenschmerzen | 0.8 |
|  |  | Zwiebelsäckchen | 2.3 |
|  |  | Was tun bei ohrenschmerzen | 0.8 |
|  |  | Schnupfen ohrenschmerzen | 0.8 |
| Ohrentropfen | 6.9 | Ohrenschmerzen | 40.4 |
| Ohrentropfen | 12.2 | Mittelohrentzündung | 43.8 |
| Ohrentropfen | 27.9 | Wala | 58.8 |
| Ohrentropfen | 39.5 | Ohrentropfen rezeptfrei | 2.6 |
|  |  | Ohrentropfen hund | 1.7 |
|  |  | Aurizon | 3.5 |
|  |  | Gehörgangsentzündung | 17.5 |
| Ohrentropfen | 41.2 | Ohrentropfen otalgan | 4.2 |
|  |  | Otalgan | 12.1 |
|  |  | Ciloxan ohrentropfen | 2.6 |
|  |  | Ciloxan | 6.5 |
|  |  | Ohrentropfen apotheke | 3.0 |
|  |  | Ohrentropfen mittelohrentzündung | 2.2 |
|  |  | Ohrentropfen ohrenschmalz | 2.1 |
|  |  | Ohrenschmalz | 40.0 |
|  |  | Aconit ohrentropfen | 2.4 |
|  |  | Ohrentropfen kinder | 1.5 |
|  |  | Wala ohrentropfen | 2.1 |
|  |  | Ohrentropfen antibiotika | 1.4 |
|  |  | Otovowen | 28.3 |
|  |  | Panotile | 5.9 |
|  |  | Aurizon ohrentropfen | 1.7 |
|  |  | Panotile ohrentropfen | 1.9 |
|  |  | Antibiotische ohrentropfen | 1.1 |
|  |  | Otobacid ohrentropfen | 1.7 |
| Otitis | 7.0 | Ohrenschmerzen | 40.4 |
|  | 11.5 | Sinusitis | 48.4 |
|  | 12.3 | Mittelohrentzündung | 43.8 |
|  | 20.2 | Amoxicillin | 42.6 |
|  | 42.4 | Otitis media | 19.7 |
|  |  | Otitis externa | 12.8 |
|  |  | Otitis externa therapie | 1.8 |
|  |  | Otitis externa maligna | 1.8 |
|  |  | Otitis media acuta | 0.9 |
|  |  | Gehörgangsentzündung | 19.0 |
|  |  | Cerumen | 13.0 |
|  |  | Mastoiditis | 12.2 |
|  |  | Pharyngitis | 29.7 |
|  |  | Cholesteatom | 17.3 |
|  |  | Tonsillitis | 37.8 |
|  |  | Otis | 6.6 |
|  |  | Cerumen obturans | 5.1 |
|  |  | Otalgie | 3.9 |
|  |  | Laryngitis | 23.5 |
|  |  | Konjunktivitis | 30.0 |
|  |  | Otitis media leitlinie | 0.4 |
|  |  | Myringitis | 3.3 |
|  |  | Rhinitis | 34.0 |
